# Hematological Abnormality, Oxidative Stress, and Genotoxicity Induction in the Greenhouse Pesticide Sprayers; Investigating the Role of NQO1 Gene Polymorphism

**DOI:** 10.3390/toxics6010013

**Published:** 2018-02-07

**Authors:** Nahid Ahmadi, Ali Mandegary, Akram Jamshidzadeh, Mohaddeseh Mohammadi-Sardoo, Mohammadreza Mohammadi-Sardo, Elham Salari, Leyla Pourgholi

**Affiliations:** 1 Department of Pharmacology and Toxicology, School of Pharmacy, Shiraz University of Medical Sciences, Akbarabad, Shiraz 71345-1583, Fars, Iran; nah.ahmadi@gmail.com (N.A.); ajamshid@sums.ac.ir (A.J.); 2 Pharmaceutics Research Center, Institute of Neuropharmacology, Kerman University of Medical Sciences, Haft-Bagh Blvd., Kerman 7616911319, Iran; 3 Department of Pharmacology and Toxicology, School of Pharmacy, Kerman University of Medical Sciences, Haft-Bagh Blvd., Kerman 7616911319, Iran; leyla.pourgholi5@gmail.com; 4 Neuroscience Research Center, Institute of Neuropharmacology, Kerman University of Medical Sciences, Jahad Blvd., Kerman 7619813159, Iran; 5 Pharmaceutical Sciences Research Center, Shiraz University of Medical Sciences, Akbarabad, Shiraz 71345-1583, Fars, Iran; 6 Department of Animal Biology, Faculty of Biological Sciences, Kharazmi University, Shahid Mofateh ST, Tehran 15719-14911, Iran; m.mohammadisardoo@yahoo.com; 7 Faculty of Medical Sciences, Jiroft University of Medical Sciences, Pasdaran Blvd, Jiroft 7861615765, Kerman, Iran; dr.mohamadi@jmu.ac.ir; 8 Deputy of Research and Technology, Jiroft University of Medical Sciences, Pasdaran Blvd, Jiroft 7861615765, Kerman, Iran; elham.salari855@yahoo.com

**Keywords:** pesticides, oxidative stress, genotoxicity, NQO1, cholinesterase, biomarkers

## Abstract

The widespread use of pesticides in agriculture represents a threat to the human populations exposed to them. In this cross-sectional study, the hematological and biochemical parameters, plasma cholinesterase (PChE) activity, oxidative stress, genotoxicity, and NAD(P)H: quinone oxidoreductase 1 (NQO1) C609T polymorphism were measured in 100 greenhouse workers occupationally exposed to pesticide mixture and 104 normal healthy controls. There was a decrease in erythrocytes (5.45%, *p* = 0.026) and hemoglobin (3.26%, *p* = 0.025), and an increase in mean corpuscular hemoglobin (3.54%, *p* = 0.013) in the exposed workers. Sprayers showed a reduction in PChE (23%) and GSH (50%) levels, and an increase in lipid peroxidation (LPO) (55%), protein carbonyl (145%), Superoxide dismutase activity (61%), and total antioxidant capacity (35%) (*p* < 0.001 for all parameters but LPO: *p* = 0.009). Genotoxicity parameters were significantly high in the exposed cases (for all parameters: *p* < 0.001 but tail length: *p* = 0.002). There was a significant correlation between oxidative stress and genotoxicity parameters, and also between these biomarkers and PChE activity. The NQO1 C609T polymorphism was not significantly associated with studied biomarkers. The findings indicate that occupational exposure to a mixture of pesticides can induce hematotoxicity, oxidative stress, and genotoxicity in greenhouse workers.

## 1. Introduction

Pesticides are among more than 1000 active ingredients that can be divided into three main classes: insecticide, herbicide, and fungicide. Despite all of the advantages of pesticides, their widespread use has released large amounts of potentially toxic substances into the environment and affected human health [1,2]. Occupational pesticide toxicity involves a large number of people, mainly in developing countries, where a significant percentage of the active population is involved in agriculture and pesticide application in inappropriate conditions, including the usage of restricted compounds and unsuitable spraying equipment [3,4]. A growing body of evidence has demonstrated the correlation between occupational exposure to pesticides and the development of a wide spectrum of pathologies, ranging from diabetes to neurological diseases and cancer [5]. Jiroft city is located in the south-eastern Kerman province and is the hub of greenhouse cultivation in Iran. The estimated amount and overall frequency of pesticide use in Jiroft is much more than is needed [6].

The alternation in some of the biological markers (e.g., hematological and biochemical parameters) might reflect an early stage in the development of a disease [7]. In recent years, increasing attention has been given to the assessment of biomarkers of human exposure to pesticides [7]. Pesticides may induce oxidative stress, leading to the generation of free radicals, accumulation of reactive oxygen species (ROS), alteration in the scavenging enzyme system, and depletion of cellular antioxidant reservoirs, finally resulting in damage to all components of the cell, including lipids, proteins, and DNA [8].

Although associations between pesticide exposure and cancer incidence in pesticide applicators are not conclusive [9,10], numerous lines of evidence suggest this association. Reactive species, including free radicals derived from oxygen, can interact with DNA and result in the mutation and strand breaks involved in cancer [11]. Hence, several genotoxic tests are introduced to evaluate the potential risk of cancer. Single-cell gel electrophoresis or the Comet assay has been established as a sensitive and versatile method for the detection of DNA strand breaks [12]. Besides, apoptotic DNA cleavage, resulting in multiples of about 180-bp oligomers, can be assessed by a DNA ladder assay.

Considering that the metabolism of some pesticides can lead to ROS generation, the role of polymorphisms in genes encoding antioxidant enzymes looks important. NAD(P)H: quinone oxidoreductase 1 (NQO1) is an important cytosolic enzyme that catalyzes the two-electron reduction of quinoids using NAD(P)H as a cofactor and detoxifies the electrophilic compounds, consequently preventing the formation of ROS [13]. However, in certain conditions, NQO1 can act as a pro-oxidant enzyme causing oxidative stress and produce intermediates that are capable of alkylating nucleophilic sites including DNA [13]. Several lines of evidence convincingly show that variability in NQO1 could have a key role in pesticide-induced oxidative stress and toxicity [14,15]. The nucleotide substitution by cytosine (C) to thymine (T) at position 609 of the NQO 1 gene, coding for proline to serine change at codon 187 (rs1800566), is essential because it directly affects the catalytic potential of the enzyme. The homozygous mutant variant (TT) and heterozygote variant genotype (CT) have shown a reduction in the enzyme activity level that may cause an additive effect on oxidative damage [16].

So, this study was designed to assess whether continuous exposure to a mixture of pesticides in sprayers engaged in intensive agriculture settings could lead to changes in oxidative stress biomarkers, genotoxicity, and hematological and biochemical parameters. Notably, the contribution of the NQO1 C609T polymorphism to the pesticide-induced oxidative stress and genotoxicity was evaluated for the first time in the present study.

## 2. Materials and Methods

### 2.1. Chemicals

The materials which were applied in this research are as follows: 2-thiobarbituric acid (TBA), pyrogallol, H2O2, FeCl3, MgCl2, Triton X-100, NaCl, Tris, EDTA, Boric acid, and DMSO from Merck KGaA (Darmstadt, Germany); 2,4,6-tripyridyl-striazine (TPTZ), acetylthiocholine iodide, dithiobis-2-nitrobenzoic acid (DTNB), ethidium bromide, and LMP agarose from Sigma-Aldrich Chemie GmbH (Darmstadt, Germany); 2,4-dinitrophenylhydrazine (DNPH) from Scharlab S.L. (Barcelona, Spain); agarose from CinnaGene (Karaj, Iran); dNTPs mix, Taq DNA polymerase, PCR buffer, DNA loading Dye, and DNA ladder from GeneAll (Seoul, Korea); and HinfI endonuclease from Jena Bioscience GmbH (Jena, Germany).

### 2.2. Study Population

The present study was conducted in an intensive agricultural area (greenhouses of Jiroft town from southeastern Iran). The study population consisted of 100 male pesticide sprayers and 104 non-agricultural males in the same environment acting as controls. The study excluded individuals who presented any pathology that could interfere with results interpretation; those who took any kind of medicine or antioxidant supplements; and those who had been exposed to any chemicals and other potentially genotoxic substances or any kinds of radiation within the last six months before blood sampling. Subjects were informed about the purpose of the study and asked to sign a consent form. The study was conducted in accordance with the Declaration of Helsinki, and the protocol was approved by Research Ethics Committee of Jiroft University of Medical Sciences on 13 June 2014 (Ir.jmu.rec.1393-17). Studied groups were surveyed by means of a detailed questionnaire covering demographic characteristics (age, height, and weight); lifestyle (smoking habit, alcohol consumption); medical and family history; and occupational features (lifetime exposure to pesticides, use of personal protective equipment, and type of pesticide used). An exposure index was calculated for each pesticide sprayer according to the following formula: hours/day × days/month × months/year × years.

### 2.3. Sample Collection

A fasting venous blood sample was collected from each subject immediately after spraying in sterile heparinized and plain vacutainers and transported to the laboratory under cold conditions. The samples were centrifuged at 3500 rpm for 15 min at 4 °C to separate serum and plasma from blood cells. Fractions were stored at −80 °C until biochemical assays and DNA extraction. The second fresh heparinized blood sampling was used for the comet assay.

### 2.4. Determination of Hematological and Biochemical Biomarkers

The hematological markers including leukocytes, erythrocytes, hematocrit, hemoglobin, mean corpuscular volume (MCV), mean corpuscular hemoglobin (MCH), mean corpuscular hemoglobin concentration (MCHC), and platelets were measured using a Mindray BC-3000 hematology analyzer (Mindray Bio-Medical Electronics Co., Shenzhen, China). The following biochemical parameters were determined by diagnostic kits from MAN Co. (Tehran, Iran) and using a Selectra Pro M automatic biochemistry analyzer (ELITech Group, Puteaux, France): fasting blood sugar (FBS); triglycerides; cholesterol; high-density lipoprotein (HDL); low-density lipoprotein (LDL); blood urea nitrogen (BUN); creatinine; aspartate aminotransferase (AST); and alanine aminotransferase (ALT). All analyses were performed in a certified clinical laboratory following routine and automatized procedures.

### 2.5. Determination of Plasma Cholinesterase Activity

Plasma cholinesterase (PChE) activity was determined by the method of Ellman et al. [17] with some modifications [18]. Briefly, the hydrolysis rate of acetylthiocholine iodide in plasma was measured at 412 nm, by the reaction with DTNB to give the yellow 5-thio-2-nitrobenzoate anion. The enzyme activity was expressed as KU/L.

### 2.6. Determination of Oxidative Stress Biomarkers

Catalase (CAT) activity was measured according to the method of Aebi [19] with some modifications [20]. Briefly, the decomposition rate of H2O2 was monitored at 240 nm after adding the diluted serum directly. Enzyme activity was expressed as Units (U). Superoxide dismutase (SOD) activity was measured according to the method of Marklund and Marklund [21] with some modifications [20]. Briefly, the inhibition of pyrogallol autoxidation by superoxide dismutase was analyzed at 420 nm for 5 min. Enzyme activity was expressed as Units (U). Lipid peroxidation (LPO) was estimated in serum as thiobarbituric acid reactive substance (TBARS) levels after incubation with TBA at a low pH and high temperature. The resulting pink complex was measured at 532 nm [22]. The serum concentration of GSH was measured spectrophotometrically at 412 nm using DTNB as the reagent [23] with minor modifications as described previously [24]. Serum protein carbonyl (PrC) levels were measured according to method described by Levine et al. [25], based on their reaction with DNPH, followed by the spectrophotometric quantification of the protein hydrazones at 365 nm. Serum total antioxidant capacity (TAC) was determined by measuring the ferric-reducing ability of plasma in the presence of TPTZ [26] with minor modifications as described previously [22]. The purple complex produced by the reaction was measured at 593 nm.

### 2.7. DNA Extraction and DNA Ladder Assay

Genomic DNA was extracted from the stored blood cells using the ‘salting out’ method [27] with some modifications [22]. DNA fragmentation was assessed by agarose gel electrophoresis [28]. The multiples of about 180-bp nucleosomal units appeared as a DNA ladder when run on the gel. The DNA solution was loaded with loading dye on 1% agarose gel containing 0.6 μg/mL ethidium bromide and electrophoresed at 90 V using Borate buffer (0.25% Boric acid, 0.04% NaOH, pH 8). The electrophoresis was stopped when the bromophenol blue dye had migrated two-thirds of the way down the gel. Appropriate DNA molecular weight markers should be included. The gel was visualized and photographed by an ultraviolet gel documentation system, EBOX VX5 (Vilber Lourmat, Marne La Vallee, France). Densitometric analysis of ladder lanes was done using ImageJ 1.50i (National Institutes of Health, Bethesda, MD, USA, 2016).

### 2.8. Comet Assay

The alkaline comet assay used here was adapted from the method previously described [29] with some modifications. Briefly, 20 μL of the blood samples was suspended in 200 μL of prewarmed LMP agarose (0.6% *w*/*v*, 37 °C), from which two 80-μL samples were dispensed directly on each microscope slide (precoated with 1% NMP agarose), and allowed to solidify under a coverslip, on ice. After gently removing the coverslip, the slides were immersed in cold, freshly-made lysing solution (2.5 M NaCl, 100 mM Na2EDTA, 10 mM Tris, 1% Triton X-100, and 10% DMSO, pH 10) at 4 °C. After an overnight lysis step, the slides were washed once with cold DW, placed in cold, freshly-made alkaline electrophoresis buffer (300 mM NaOH, 1 mM Na2-EDTA, pH > 13) for 30 min, and then electrophoresed at 25 V/300 mA for 30 min. The slides were neutralized with neutralization buffer (0.4 M Tris, pH 7.5) thrice for 5 min and stained with 2 μg/mL ethidium bromide. All the steps were carried out under dimmed light. The gels were visualized by an Eclipse Ti-U fluorescent microscope (Nikon Corporation, Tokyo, Japan) at 400× magnification equipped with a DS-Ri2 camera (Nikon Corporation, Tokyo, Japan). Fifty to a hundred comets per individual were randomly selected and analyzed using CASPLab 1.2.2 (Institute of Theoretical Physics, Zurich, Switzerland, 2003). Results were expressed as tail length (distance from the right border of the head area to the end of the tail, in pixels), tail DNA% (percent of DNA in the tail), tail moment (tail DNA% × tail length, in arbitrary units), and olive tail moment (tail DNA% × distance between the center of gravity of DNA in the tail and the head, in arbitrary units).

### 2.9. NQO1 Genotyping

The polymorphic NQO1 (rs1800566) gene was determined using the polymerase chain reaction (PCR)–restriction fragment length polymorphism (RFLP) method, as previously published [16]. Briefly, PCR was performed with the 25 μL reaction mixture containing 1 μL of DNA, 10 μM of each primer, 10 mM dNTPs, 25 mM of MgCl2, 5 U/μL of Taq polymerase, and 10× buffer. The amplification of the 273-bp fragment of the NQO1 gene was carried out in a thermocycler, PEQStar (PEQLAB Biotechnologie GmbH, Erlangen, Germany), using a pair of sense and antisense primers including 5′-AGTGG CATTC TGCAT TTCTGTG-3′ and 5′-GATGG ACTTG CCCAAG TGATG-3′. The initial denaturation step was performed at 95 °C for 5 min, followed by 33 cycles consisting of three steps: 95 °C for 28 s, 62 °C for 30 s, and 72 °C for 28 s. The final elongation was done at 72 °C for 5 min. The products were incubated with HinfI endonuclease (1U) for 16 h at 37 °C. After the digestion step, the PCR products and digested products were separated by 2% agarose gel electrophoresis and stained by ethidium bromide. The genotypes were determined by the pattern on the digested bands visualized in the ultraviolet gel documentation system (188 and 85 bp bands: CC genotype; 151 and 85 bp bands: TT genotype; 188, 151, and 85 bp bands: CT genotype).

### 2.10. Statistical Analysis

The normality of continuous data was assessed using statistical tests (Kolmogorov–Smirnov test) and visual inspection (p–p plots and histogram). Data were expressed as a mean ± SD for normally distributed variables or median and interquartile ranges (IQR) for non-normal data. The differences between the quantitative variables of the two study groups were tested by the Student’s-sample *t*-test and Mann–Whitney U test for normally and non-normally distributed data, respectively. The Chi-square test was used to compare frequency counts between study groups. Multiple linear regression analysis was used to estimate the effect of several variables on plasma cholinesterase activity and oxidative stress biomarkers as dependent variables in two models. The first model was adjusted for the following predictors: pesticide exposure, age, body mass index (BMI), smoking status, and NQO1 polymorphisms. In the second model, only pesticide sprayers were included in the analysis and adjustment was made for the same predictors plus the following occupational variables: pesticide exposure index and utilization of personal protective equipment (PPE). The associations between plasma cholinesterase activity and oxidative stress biomarkers were evaluated appropriately by Pearson’s correlation and Spearman’s rank-order correlation. Two tailed p-values less than 0.05 were considered to indicate statistical significance. All statistical analyses were conducted with SPSS software (SPSS for Windows, Version 16.0., SPSS Inc. Chicago, IL, USA, 2007).

## 3. Results

Demographic characteristics of controls and pesticide sprayers are shown in Table 1. There were no significant differences between the two groups in regard to age, BMI, smoking status, and NQO1 polymorphisms. Average duration of exposure in years and average hourly exposure per day were 5.29 and 4.64, respectively.

Pesticide features used by exposed subjects are summarized in Table 2. Most of the pesticides have been classified as carcinogenic by the US Environmental Protection Agency [30] and hazardous by the World Health Organization [31], but are not yet listed by the International Agency for Research on Cancer [32].

The mean value of hematological and biochemical parameters, as well as the number of subjects having biomarkers outside the reference value in both groups, are reported in Table 3 and Table 4, respectively. The significant increase of MCH (3.54%, *p* = 0.013) and decrease of the erythrocytes count (5.45%, *p* = 0.026) and hemoglobin (3.26%, *p* = 0.025) in the exposed population relative to the control group was observed. The significant differences in the number of subjects with an erythrocytes count < 4.5 × 10^6^/μL (*p* = 0.001), hemoglobin < 14 g/dL (*p* = 0.034), hematocrit < 41.5% (*p* < 0.001), FBS > 109 mg/dL (*p* = 0.002), LDL > 130 mg/dL (*p* = 0.027), BUN > 40 mg/dL (*p* = 0.010), and ALT > 40 U/L (*p* = 0.006) were found between study groups. No significant differences were found in the remaining hematological and biochemical biomarkers.

**Figure 1:**
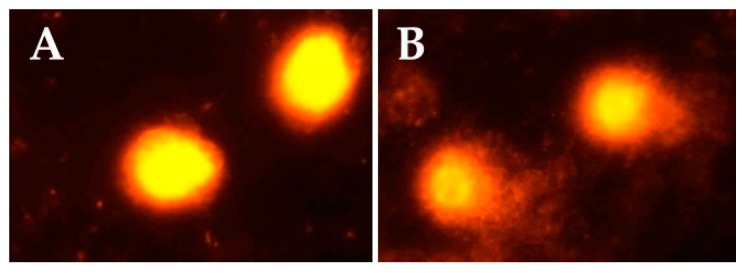
Evaluation of DNA damage by the comet assay in controls (**A**) and exposed subjects (**B**). Ethidium bromide-stained comets were visualized by a fluorescent microscope at 400× magnification.

A comparison of the levels of plasma cholinesterase activity, serum oxidative stress biomarkers, and genotoxicity parameters between the pesticide sprayers and controls is shown in Table 5. The exposed group recorded a significant reduction in the activity of PChE (23%) versus the control group (*p* < 0.001). Statistically significant differences were observed for all oxidative stress parameters between the two studied groups (for all parameters: *p* < 0.001 but LPO: *p* = 0.009), with the exception of CAT. A significant increase in the levels of SOD (61%), LPO (55%), PrC (145%), and TAC (35%) was found in pesticide sprayers, while GSH (50%) showed a significantly decreased level with respect to the controls. A significant increase was observed in all genotoxicity parameters in the exposed group (for all parameters: *p* < 0.001 but tail length: *p* = 0.002). Figure 1 indicates representative comet assay images in the control and exposed groups. While control samples displayed mostly intact cells with undamaged DNA (without comet tail), samples of sprayers showed cells with DNA damage and comet tails.

Multivariate linear regression was performed to analyze the significant differences of plasma cholinesterase activity, oxidative stress biomarkers, and genotoxicity parameters between the two studied groups, while adjusting for explanatory variables including age, BMI, smoking status, and NQO1 polymorphisms. Table 6 presents the effect of pesticide exposure on the biomarker levels by considering explanatory variables. The second model of multivariate analysis was only conducted in the pesticide-exposed group in which, in addition to the abovementioned explanatory variables, the pesticide exposure index and utilization of PPE were considered. There was no significant association between measured biomarkers and occupational explanatory variables, with the exception of a direct association between the pesticide exposure index and levels of TAC and GSH (β = 0.27, *p* = 0.02 and β = 0.24, *p* = 0.04, respectively). The Student’s-sample *t*-test and Mann–Whitney U test were also conducted appropriately to compare plasma cholinesterase activity, oxidative stress biomarkers, and genotoxicity parameters between two groups of CC and clumped CT+TT (as carriers of T allele) genotypes of NQO1 in study groups, and no significant differences were found.

The Pearson’s correlation and Spearman’s rank-order correlation were run appropriately to determine the relationship between oxidative stress and genotoxicity parameters (Table 7), and also their association with PChE. PChE was found to be inversely correlated with SOD (r_s_ = −0.491), PrC (r_s_ = −0.492), and TAC (r_s_ = −0.371), but directly associated with CAT (r_s_ = 0.310), LPO (r_s_ = 0.283), and GSH (r_s_ = 0.416), which was statistically significant for all parameters. The percentage of integrated density of 180 (r_s_ = −0.274), 360 (r_s_ = −0.267) and 540 bp (r_s_ = −0.286) bands and Tail DNA (r_s_ = −0.217) also showed an inverse association with PChE, which was statistically significant for all parameters with the exception of Tail DNA.

## 4. Discussion

The present study was conducted to evaluate possible changes in hematological and biochemical parameters, plasma cholinesterase activity, and oxidative stress biomarkers, in a particular group of pesticide sprayers occupationally exposed to mixtures of these chemicals for a number of years.

The result of this study showed some hematological and biochemical abnormalities in the pesticide-exposed population. In contrast to previous studies that reported no significant changes in erythrocyte count, hemoglobin, and MCH levels [7,33,34], our results demonstrated a higher level of MCH and lower levels of erythrocytes and hemoglobin in the sprayer population. These outcomes, plus the existence of more subjects with an erythrocytes count < 4.5 × 10^6^/μL, hemoglobin < 14g/dL, and hematocrit < 41.5% in the exposed group, could be the result of the binding of organophosphate pesticides on iron, and impairment in the synthesis of heme and hemoglobin [35,36]. As shown in earlier controversial reports [7,33,34,37,38,39], the present significant difference between two groups in the number of individuals with abnormal FBS (>109 mg/dL), LDL (>130 mg/dL), BUN (>40 mg/dL), and ALT (>40 U/L), recommends extended research in the future to explore the effect of pesticides on glucose and lipids’ metabolism, in addition to the function of the kidney and liver. The disrupting effects of pesticides, in particular organophosphates, on glucose homeostasis could be linked to oxidative damages, inflammatory cytokines, a lipotoxic effect, and subsequently insulin resistance [40]. Pesticides may also induce impairments in the metabolism of carbohydrates, lipids, and proteins through oxidative stress [41].

Plasma cholinesterase, as a biomarker of exposure to anticholinesterase pesticides, was significantly decreased in the pesticide appliers, relative to the control group. The type of pesticide used by workers and especially the use of organophosphates and/or carbamates, can be related to an inhibition of cholinesterase activity. Our results support the earlier findings that reported significant reductions in cholinesterase activity in the pesticide-exposed subjects [7,35,38,42,43,44]. However, Sudjaroen1 et al. [34] reported that cholinesterase activity was not significantly inhibited in the population exposed to a mixture of pesticides. It is worth mentioning that blood cholinesterase inhibition is one biochemical measure used by the U.S. Environmental Protection Agency for risk assessments of organophosphate and carbamate pesticides [45].

The present study showed that pesticide-exposed sprayers had overall altered levels of oxidative stress biomarkers. According to most previous studies, these findings indicate that pesticides disturb the redox status and so have harmful effects on human health. Pesticides may induce oxidative stress via several mechanisms, such as an enhanced generation of highly reactive molecules (e.g., ROS) and/or altered capacity of the antioxidant system of the body (e.g., CAT, SOD, and GSH). This is reflected by the increase in protein carbonyl contents as an excellent marker of protein oxidation and lipid peroxidation [46]. This study showed that exposed subjects presented significantly higher levels of LPO and PrC with respect to the controls, in agreement with earlier studies [37,42,47,48,49,50,51,52,53,54]. Nevertheless, other studies indicated no difference in LPO between exposed and control groups [39,44,55]. In accordance with some reports [37,44,47,52], the GSH level declined in pesticide sprayers, which may be due to the increased utilization for detoxification of reactive molecules such as ROS induced by pesticide exposure. However, some other studies showed no changes in the GSH content in exposed subjects [42,51]. Unlike the reports of Mecdad et al. [49], there was a significant increase in TAC in the pesticide-exposed group that could be due to the adaptive response to the generated free radicals [56]. The elevated activity of SOD in sprayers, as reported by Prakasam et al. [47], may reflect an activation of the compensatory mechanism through the pesticide-induced overproduction of reactive species. However, this finding is different to the previous studies, which showed a reduction in the SOD activity in the exposed group [44,57,58,59]. DNA fragmentation into fragments of multiples of about 180 bp, close to the oligonucleosome-sized fragments observed during apoptosis, was increased in the exposed group. In agreement with some investigations [60,61,62], these results suggested that pesticide exposure could result in typical chromatin DNA damage or DNA laddering, characteristic of cells during apoptosis. The cleavage of chromatin DNA into inter-nucleosomal fragments has a key role in the agrochemical-induced genotoxicity. The results of the Comet assay presented in this study, together with earlier studies [38,53,54,63,64,65,66], revealed that the occupational exposure to pesticides could induce DNA damage. However, Zepeda-Arce et al. [55] reported no statistical differences in the measured genotoxic parameters among the study groups. These findings were further confirmed by the multivariate linear regression analysis while adjusting for factors including age, BMI, smoking status, and NQO1 polymorphism, showing that exposure to pesticides as a determinant factor was strongly associated with plasma cholinesterase activity, oxidative stress, and genotoxicity biomarkers. Lopez et al. [57] also reported that exposure to pesticides was a determinant in the alternation of SOD, independent of age, BMI, and smoking habit. The other investigation showed that individuals in the pesticide exposure group (regardless of gene-environmental factors) had an increased DNA tail moment, relative to controls [64]. Multivariate linear regression in the exposed group showed a significant direct association between the pesticide exposure index (hours/day × days/month × months/year × years) and levels of TAC and GSH that may be due to the adaptive response to adverse effects of pesticide exposure. Madani et al. [37] and Rekhadevi et al. [54] showed that the duration of pesticide use was significantly associated with the induction of oxidative stress and genotoxicity. However, another study showed no significant correlation between oxidative stress biomarkers and years of exposure [58]. Singh et al. [65] also reported no significant difference in DNA tail moment on the basis of duration of exposure to pesticides, but another study showed that the amount of DNA tail moment correlated with the extent of pesticide exposure [64]. There was no significant association between the measured biomarkers and PPE in the present study. This could be because sprayers do not always pay enough attention to the renewal or cleaning of their protective equipment, leading to the loss of the effective protection afforded by them [38]. The genotoxicity findings are also in agreement with research findings in extant literature, which reported that Comet assay values showed no significant difference between those who used complete protective equipment and those who did not [38,63]. On the other hand, some studies showed a significant relationship between the utilization of PPE during the spraying of pesticides and alternation of cholinesterase activity and oxidative stress biomarkers [37,57,67].

No association was found between the measured biomarkers and NQO1 polymorphism rs1800566. Nonetheless, to the best of our knowledge, the present study is the first to assess the role of NQO1 polymorphism in oxidative stress and genotoxicity induced in cases with long-term pesticide exposure, and future investigations are required to obtain more conclusive data on NQO1 and pesticide-induced oxidative stress and DNA damage.

The Spearman’s correlation analysis showed significant correlations between all oxidative stress biomarkers with PChE activity as a biomarker of exposure to anticholinesterase pesticides in the exposed group. In accordance with a previous study [66], an inverse association between PChE activity and DNA damage indices was found in the present study. In fact, some authors consider that cholinesterase inhibition induces cholinergic hyperactivity, initiating the accumulation of free radicals and leading to the damage of cellular components and pesticide-induced toxicity [68,69]. In contrast to the report of Mecdad et al. [49], this study showed that the inhibition in PChE activity correlated with the increase in TAC and decrease in GSH levels. These findings may be due to the adaptive and defensive responses to the generated free radicals induced by pesticide exposure, respectively. In accordance with a previous study [58], CAT activity was directly related to PChE activity in the present study. A mechanistic explanation for this finding might be the inhibition of thiol groups of CAT by the generated oxygen free radicals induced by pesticide exposure [70]. SOD presented an inverse association with PChE activity, reflecting an activation of the compensatory mechanism induced by the overproduction of reactive species, whereas other studies showed contradictory relations between these two parameters [55,58]. The reduction in PChE activity was associated with a higher level of PrC, which was the biomarker of pesticide exposure-induced oxidative stress damage. In contrast to previous studies [44,48,66], the present study showed that the decrease in PChE activity was associated with a lower level of TBARS. The significant relationship between oxidative stress and genotoxicity biomarkers, as shown in the results reported by Zepeda-Arce et al. [55] and Jacobsen-Pereira et al. [66], shows that pesticide exposure could lead to DNA damage and apoptosis by inducing oxidative stress via the generation of ROS and alternation of the capacity of the antioxidant system.

Despite the several strengths of this study including a case-control design, extensive multivariate statistical analysis, evaluation of correlations among different biomarkers, and taking into account the role of variability in an important gene, our study should also be evaluated in light of its weaknesses. We did not manage to quantify the pesticide concentration. In addition, by using a cross-sectional design, we were unable to directly account for historical effects on the studies’ biomarkers.

## 5. Conclusions

The present study revealed that long-term exposure to a mixture of pesticides could disrupt the balance between the production of free radicals and antioxidant defenses, resulting in DNA damage. Various biological markers such as plasma cholinesterase activity, oxidative stress, and genotoxicity parameters can be applied to monitor the early adverse effects of pesticides on the health of agricultural workers. This study, in conjunction with several biomonitoring studies, highlights the need to properly evaluate and control the potential health effects of exposure to mixtures of toxic chemicals among workers employed in intensive agriculture settings.

## Figures and Tables

**Table 1:** Demographic characteristics of the study groups.

Characteristics	Controls (*n* = 104)	Pesticide Sprayers (*n* = 100)	*p*-Value
Age (years)	36.89 ± 13.01	33.64 ± 12.36	0.070
BMI (kg/m^2^)	23.64 ± 4.34	22.76 ± 3.49	0.123
Smoker (n)	11	19	0.094
NQO1 genotypes			
CC	46	47	0.884
CT+TT	45	50	
Use of PPE (n)	NA	44	NA
Years of exposure	NA	5.29 ± 4.34	NA
Hours of exposure in day	NA	4.64 ± 3.09	NA

Data are expressed as numbers of individuals or means ± SD and comparisons were made by using the Chi-square test or Student’s-sample *t*-test, respectively. PPE: personal protective equipment; BMI: body mass index; NA: not applicable.

**Table 2:** List of pesticides used by the exposed subjects.

Pesticide	Common Name	Chemical Class	IARC	US EPA	WHO
Fungicides	Mancozeb	Dithiocarbamate	NL	B	U
	Carbendazim	Benzimidazole	NL	C	U
	Metalaxyl	Benzenoid	NL	E	II
	Benomyl	Benzimidazole	NL	C	U
	Tebuconazole	Triazole	NL	C	II
	Trifloxystrobin	Strobilurin	NL	Not Likely	U
Insecticides	Abamectin	Macrocyclic Lactone	NL	E	NL
	Diazinon	Organophosphate	2A	Not likely	II
	Imidacloprid	Chloronicotinyl	NL	E	II
	Thiamethoxam	Neonicotinoid	NL	Not likely	NL
	Pyridaben	Pyridazinone	NL	E	II
	Fenpyroximate	Pyrazole	NL	Not likely	II
	Deltamethrin	Pyrethroid ester	3	Not Likely	II
	Thiacloprid	Neonicotinoid	NL	Likely	II

IARC (2017) classification: 2A—probably carcinogenic to humans; 3—not classifiable as to its carcinogenicity to humans. US EPA (2016) classification: B—probable human carcinogen; C—possible human carcinogen; E—evidence of non-carcinogenicity for humans. WHO (2009) classification: II—moderately hazardous; U—unlikely to present acute hazard in normal use. NL: not listed.

**Table 3:** Hematological biomarkers in study groups.

Biomarkers	Controls	Pesticide Sprayers	Reference Values	*p*-Value
Leukocyte count (×10^3^/μL)	6.9 ± 2.0	6.7 ± 1.7	4.4–11	0.441
Leukocyte count < 4.4×10^3^ (n)	9	8		0.534
Erythrocytes count (×10^6^/μL)	5.5 ± 0.6	5.2 ± 0.9	4.5–6.5	**0.026**
Erythrocytes count < 4.5 × 10^6^ (n)	3	16		**0.001**
Hemoglobin (g/dL)	15.3 ± 1.6	14.8 ± 1.7	14–17.5	**0.025**
Hemoglobin < 14 (n)	22	34		**0.034**
Hematocrit (%)	44.2 ± 3.9	42.9 ± 6.1	41.5–50.4	0.085
Hematocrit < 41.5 (n)	21	43		**<0.001**
MCV (fL)	80.4 ± 9.4	81.8 ± 6.6	80–96	0.215
MCV < 80 (n)	33	30		0.418
MCH (pg)	28.2 ± 3.05	29.2 ± 2.5	27–33.2	**0.013**
MCH < 27 (n)	25	19		0.218
MCHC (g/dL)	34.5 ± 1.3	34.8 ± 1.6	33.4–35.5	0.125
MCHC < 33.4 (n)	19	16		0.380
Platelets (×10^3^/μL)	241.4 ± 64.2	236.2 ± 57.9	150–450	0.546
Platelets < 150 × 10^3^ (n)	5	3		0.377

Data are expressed as numbers of individuals or means ± SD and comparisons were made by using the Chi-square test or Student’s-sample *t*-test, respectively. The bold *p*-values represent the significant differences between study groups. MCV: mean corpuscular volume, MCH: mean corpuscular hemoglobin, MCHC: mean corpuscular hemoglobin concentration.

**Table 4:** Biochemistry biomarkers in study groups.

Biomarkers	Controls	Pesticide Sprayers	Reference Values	*p*-Value
FBS (mg/dL)	93.9 ± 29.1	96.9 ± 16.0	60–109	0.356
FBS > 109 (n)	6	20		**0.002**
Triglycerides (mg/dL)	133.0 ± 52.9	145.3 ± 79.8	60–170	0.204
Triglycerides > 170 (n)	20	30		0.060
Cholesterol (mg/dL)	168.8 ± 33.3	163.4 ± 29.2	130–200	0.227
Cholesterol > 200 (n)	19	12		0.133
HDL (mg/dL)	44.6 ± 8.9	42.8 ± 9.3	30–55	0.147
HDL < 30 (n)	9	7		0.430
LDL (mg/dL)	99.5 ± 35.9	90.9 ± 25.2	100–130	0.050
LDL > 130 (n)	16	6		**0.027**
BUN (mg/dL)	29.8 ± 6.2	30.8 ± 9.6	20–40	0.382
BUN > 40 (n)	6	17		**0.010**
Creatinine (mg/dL)	0.9 ± 0.13	0.8 ± 0.3	0.5–1.3	0.161
Creatinine > 1.3 (n)	0	1		0.493
AST (U/L)	28.1010.5	28.2 ± 11.7	20–40	0.957
AST > 40 (n)	12	8		0.263
ALT (U/L)	33.2 ± 15.6	30.7 ± 13.4	10–40	0.211
ALT > 40 (n)	25	10		**0.006**

Data are expressed as numbers of individuals or means ± SD and comparisons were made by using the Chi-square test or Student’s-sample *t*-test, respectively. The bold *p*-values represent the significant differences between study groups. FBS: fasting blood sugar, HDL: high-density lipoprotein, LDL: low-density lipoprotein, BUN: blood urea nitrogen, AST: aspartate aminotransferase, ALT: alanine aminotransferase.

**Table 5:** Plasma cholinesterase activity, serum oxidative stress biomarkers, and genotoxicity parameters in controls and pesticide sprayers.

Biomarkers	Controls	Pesticide Sprayers	*p*-Value
PChE (KU/L)	0.30 ± 0.07	0.23 ± 0.10	**<0.001**
CAT (U)	64.11 (32.10–96.63)	64.12 (49.45–103.92)	0.185
SOD (U)	16.81 (14.22–18.10)	27.91 (15.52–36.63)	**<0.001**
TBARS (μM)	0.18 (0.12–0.28)	0.24(0.13–0.49)	**0.009**
GSH (μM)	81.75 (71.35–97.74)	42.04 (41.33–43.55)	**<0.001**
PrC (mM)	2.20 (1.58–2.97)	5.45 (2.96–8.14)	**<0.001**
TAC (mM)	0.85 (0.73–0.96)	1.07 (0.87–1.37)	**<0.001**
Density of 180 bp band (%)	4.55 ± 0.40	4.82 ± 0.43	**<0.001**
Density of 360 bp band (%)	3.96 (3.78–4.28)	4.22 (4.01–4.64)	**<0.001**
Density of 540 bp band (%)	3.50 (3.29–3.75)	3.70(3.42–4.13)	**<0.001**
Tail length (pixels)	3.03 (3.01–3.08)	3.07 (3.03–3.20)	**0.002**
Tail DNA (%)	1.06 ± 0.29	1.60 ± 0.43	**<0.001**
Tail moment (AU)	0.03 (0.03–0.04)	0.05 (0.04–0.08)	**<0.001**
Olive Tail moment (AU)	0.25 (0.21–0.27)	0.35 (0.31–0.39)	**<0.001**

Data are expressed as median (IQR) or means ± SD and comparisons were made by using the Mann–Whitney U test or Student’s-sample *t*-test, respectively. The bold *p*-values represent the significant differences between study groups. PChE: plasma cholinesterase, CAT: catalase, SOD: superoxide dismutase, TBARS: thiobarbituric acid reactive substances, GSH: glutathione, PrC: protein carbonyl, TAC: total antioxidant capacity, AU: arbitrary unit.

**Table 6:** Association between pesticide exposure and plasma cholinesterase activity, oxidative stress biomarkers, and genotoxicity parameters in the study population.

Biomarkers	B (CI)	β	*p* Value
PChE	−0.06 (−0.09, −0.04)	−0.35	**<0.001**
CAT	1.61 (−19.49, 22.71)	0.01	0.88
SOD	10.55 (7.79, 13.31)	0.51	**<0.001**
TBARS	0.12 (0.04, 0.19)	0.24	**0.002**
GSH	−47.44 (−56, −38.88)	−0.65	**<0.001**
PrC	3.41 (2.65, 4.18)	0.57	**<0.001**
TAC	0.3 (0.21, 0.38)	0.47	**<0.001**
Density of 180 bp band	0.27 (0.15, 0.40)	0.31	**<0.001**
Density of 360 bp band	0.30 (0.17, 0.43)	0.33	**<0.001**
Density of 540 bp band	0.36 (0.20, 0.52)	0.33	**<0.001**
Tail length	0.06 (0.02, 0.11)	0.28	**0.009**
Tail DNA	0.62 (0.47, 0.77)	0.67	**<0.001**
Tail moment	0.02 (0.01, 0.04)	0.46	**<0.001**
Olive Tail moment	0.13 (0.03, 0.22)	0.76	**<0.001**

B: Partial regression coefficient, CI: Confidence interval, β: Standardized coefficient, NS: Non-significant model. Biomarkers were adjusted for pesticide exposure (0: controls; 1: pesticide sprayers), age, BMI, smoking status and NQO1 genotypes by using Multiple linear regression. The bold *p*-values represent the significant associations between pesticide exposure and measured biomarkers. PChE: plasma cholinesterase, CAT: catalase, SOD: superoxide dismutase, TBARS: thiobarbituric acid reactive substances, GSH: glutathione, PrC: protein carbonyl, TAC: total antioxidant capacity.

**Table 7:** Overall correlations between oxidative stress biomarkers and genotoxicity parameters.

Biomarkers	CAT	SOD	TBARS	GSH	PrC	TAC
Density of 180 bp band	−0.049	0.111	0.083	−0.260 **	0.256 **	0.233 **
Density of 360 bp band	−0.141	0.187 **	−0.049	−0.248 **	0.315 **	0.246 **
Density of 540 bp band	−0.184 *	0.204 **	−0.138	−0.233 **	0.338 **	0.304 **
Tail length	0.185	0.009	0.129	−0.324 **	0.205 *	0.39
Tail DNA	0.185	−0.001	0.250 *	−0.550 **	0.287 **	0.090
Tail moment	0.222 *	−0.007	0.173	−0.482 **	0.271 **	0.090
Olive Tail moment	0.223 *	0.0003	0.282 **	−0.629 **	0.315 **	0.148

* *p* < 0.05 and ** *p* < 0.001 using Spearman’s rank-order correlation. CAT: catalase, SOD: superoxide dismutase, TBARS: thiobarbituric acid reactive substances, GSH: glutathione, PrC: protein carbonyl, TAC: total antioxidant capacity.
